# The influence of gender on ICU admittance

**DOI:** 10.1186/s13049-015-0191-2

**Published:** 2015-12-24

**Authors:** Emma Larsson, Erik Zettersten, Gabriella Jäderling, Anna Ohlsson, Max Bell

**Affiliations:** Department of Anaesthesia, Surgical Services and Intensive Care (ANOPIVA) F2:00, Karolinska University Hospital Solna, 171 76 Stockholm, Sweden; Department of Physiology and Pharmacology, Section of Anaesthesia and Intensive Care Medicine, Karolinska Institutet, Stockholm, Sweden

## Abstract

**Background:**

We assume that critically ill patients are admitted to an intensive care unit (ICU) based on their illness severity coupled with their co-morbidities. Patient attributes such as religion, nationality, socioeconomic class or gender are not relevant in this setting. We aimed to explore the association of patient gender with admission to the ICU amongst hospital physicians working in Sweden.

**Methods:**

Primary outcome assessed was gender bias among respondents. Two different versions of an online survey, with eight patient cases, were sent to physicians in Sweden who within their field of specialty meet patients that could be eligible for intensive care. The versions of the survey were identical except that *the patient gender in each case was exchanged between the two surveys*. Depending on the respondent’s birthday (odd or even number) they were directed to one of the two surveys. At the end of each case the respondent was asked to answer if they thought that the patient needed ICU care, yes or no. The respondents were not told in advance about the design of the survey. The respondents were also asked to state their age, sex, field of specialty, size of hospital and title.

**Results:**

Of 1426 respondents, 679 and 747 answered survey 1 and 2, respectively. Overall, there were no significant differences in willingness to admit in between cases describing a man or woman in the physician responses.

**Discussion:**

Anesthesiology/intensive care physicians more often choose to admit patients to the ICU compared to all other specialties. Female physicians tended to be more willing to admit patients, regardless of patient gender, than their male counterparts.

**Conclusions:**

Using a survey, with eight cases differing only with regards to the gender of the patient, we demonstrate an absence of a gender bias among Swedish hospital physicians.

**Electronic supplementary material:**

The online version of this article (doi:10.1186/s13049-015-0191-2) contains supplementary material, which is available to authorized users.

## Background

We assume that critically ill patients are admitted to an intensive care unit (ICU) based on their present illness severity coupled with their co-morbidities. Patient attributes such as religion, nationality, socioeconomic class or gender are not relevant in this setting.

Challenging this, Valentin et al. in Austria showed that men more than women were admitted to ICU even when illness severity was greater in women [1]. In one study from Sweden, 60 % of all ICU care was spent on men but they did have a higher severity of illness compared to women [2]. Gender bias has been described in other clinical settings. In 2012 Gomez and co-workers showed that among severely injured patients fewer women were directed to a trauma centre by either emergency medical service personnel or by physicians working in non-trauma facilities compared to men with comparable injury severity [3].

In managing coronary artery disease gender bias has been extensively described. One study with data from Finland, Italy and Argentina showed women to receive less aggressive evidence-based drug therapies for secondary prevention than men, with women less likely to receive aspirin, statins, and β-blockers at discharge despite presenting with higher risk profiles and demonstrating higher in-hospital and 6-month mortality [4]. Johnston et al. found that female sex was associated with less use of reperfusion (fibrinolysis or primary percutaneous coronary intervention) during ST elevation myocardial infarction in both Sweden and Canada [5].

In the light of the gender inequalities described above, the present study examines if there is a gender bias in admitting patients to the ICU. Our experiment aimed to test if the threshold to intensive care differed between male and female patients. Secondly, we assess if physicians make different choices based on their specialty and sex.

## Methods

### Design and study population

Two different versions of an online survey (Textalk, Mölndal, Sweden), with eight patient cases, were sent to physicians in Sweden who within their field of specialty meet patients that could be eligible for intensive care. During a 2 month period, from January to March, emails containing a link to the survey were sent to doctors working in Sweden. Three reminders, also in form of emails were distributed during this period. On the online study form, each respondent gave their informed consent. The full survey, translated, is available in Additional file 1. The cases were based on real life patients, but tweaked in order to be controversial and designed to be open for debate whether to admit or not to admit to the ICU. We limited the study base to physicians working at any one of the fifteen hospitals in Sweden reporting most cases to the Swedish Intensive care Register (SIR) (Additional file 1: Table S2). The versions of the survey were identical except that *the patient gender in each case was exchanged between the two surveys* (Additional file 1: Table S1). Depending on the respondent’s birthday (odd or even number), they were directed to one of the two surveys. At the end of each case the respondent was asked to answer if they thought that the patient needed ICU care, yes or no. The respondents were not told in advance about the design of the survey. The respondents were also asked to state their age (grouped as 20–29, 30–39, 40–49, 50–59, 60–69 and 70–79), sex, field of specialty, size of hospital and title.

### Statistical analysis

The following comparisons were made regarding the respondents’ willingness to admit to intensive care:

- female vs. male gender of the patient case
- female vs. male physician respondents, regardless of the gender of the patient
- anesthesiology/intensive care respondents vs. other respondents, regardless of the gender of the patient case

Differences in proportions were compared by Chi-square test. All tests were two-sided. A *p*-value of <0.05 was considered as statistically significant. Data analysis was performed using IBM SPSS Statistics 22.0 (SPSS Statistics IBM, Armonk, New York).

### Ethical approval

The Regional Research Ethics Committee in Stockholm approved the study.

## Results

**Fig. 1:**
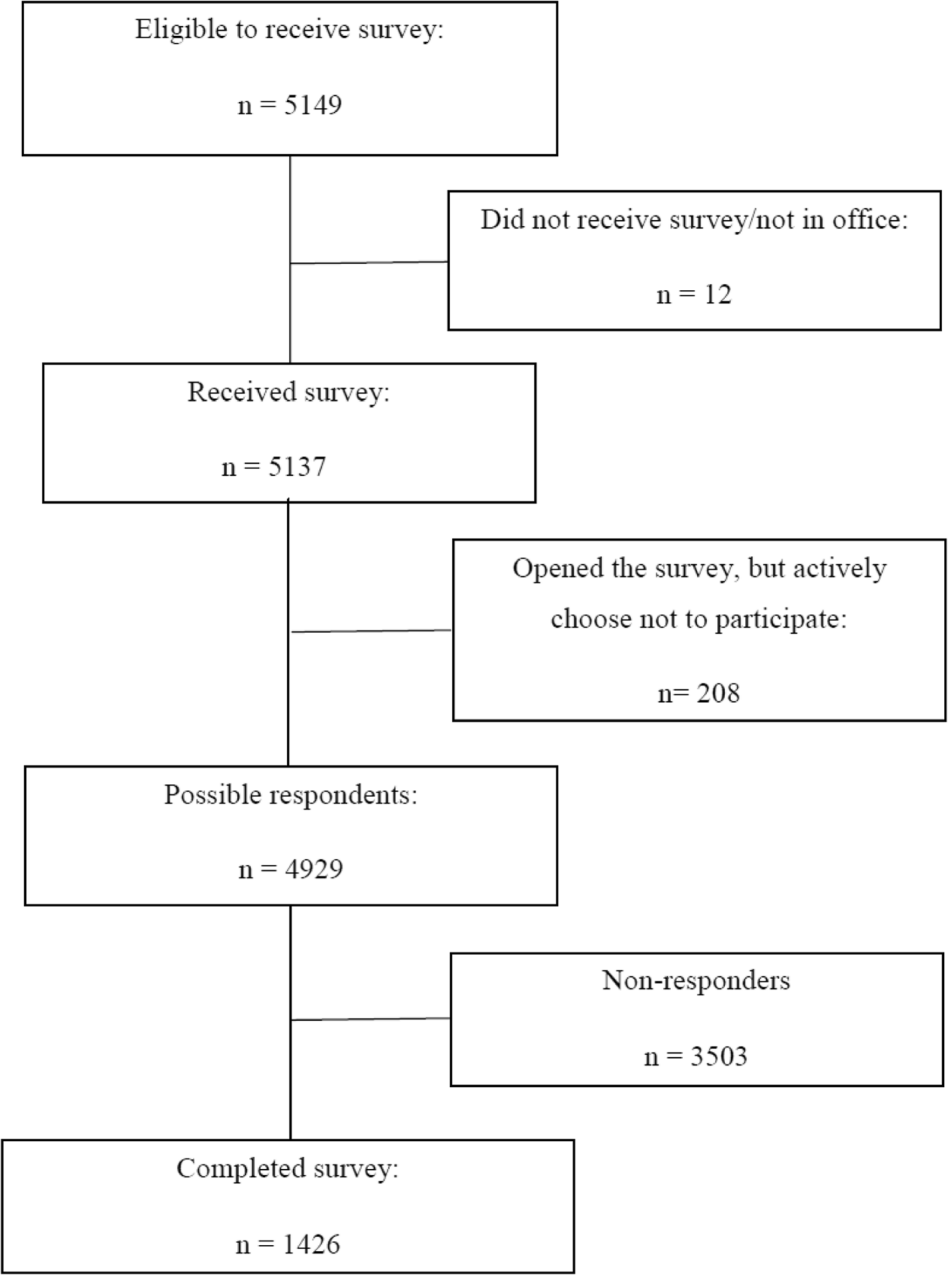
Flow chart

During the time frame described in the methods we received 1426 responses overall (Fig 1). This represents a response rate around 30 %. The demographic characteristics of the respondents are presented in Table 1, showing an equal distribution between the two surveys. Since more people are born on an odd-numbered date, survey 2 has more responders. There were more men than women completing the survey, and the majority of answers came from physicians practicing in regional hospitals. Internists and the anesthesiology/intensive care physicians dominate at slightly under and over 20 % of the answers. Physicians between 30–39 years stood for around 40 % of survey answers. In the supplementary materials the survey with cases and questions can be accessed. We found no significant differences between male and female case descriptions in the willingness to admit patients to intensive care (Table 2). In no case was there complete agreement among the physicians on whether to admit the patient or not.

**Table 1:** Demographic data of respondents

	Survey 1 *n = 679*	Survey 2 *n = 747*
*Gender (n, %)*		
Women	298 (43.9 %)	320 (42.8 %)
Men	381 (56.1 %)	427 (57.2 %)
*Age (years, %)*		
20–29	31 (4.6 %)	23 (3.1 %)
30–39	279 (41.1 %)	288 (38.6 %)
40–49	188 (27.7 %)	229 (30.7 %)
50–59	108 (15.9 %)	126 (16.9 %)
60–69	71 (10.5 %)	78 (10.4 %)
70-79	2 (0.3 %)	3 (0.4 %)
*Type of Hospital (n, %)*		
Regional Hospital	495 (72.9 %)	542 (72.6 %)
Central Hospital	151 (22.2 %)	173 (23.2 %)
Rural Hospital	22 (3.2 %)	21 (2.8 %)
Not given	11 (1.6 %)	11 (1.5 %)
*Title (n,%)*		
Resident	236 (34.8 %)	247 (33.1 %)
Board certified specialist	148 (21.8 %)	174 (23.3 %)
Assistant senior physician	37 (5.4 %)	39 (5.2 %)
Senior Physician	258 (38.0 %)	287 (38.4 %)
*Specialty (n,%)*		
Emergency medicine	30 (4.4 %)	32 (4.3 %)
Anesthesiology /Intensive Care	166 (24.4 %)	166 (22.2 %)
Gynecology	52 (7.7 %)	47 (6.3 %)
Infectious diseases	44 (6.5 %)	64 (8.6 %)
Cardiology	44 (6.5 %)	46 (6.2 %)
General surgery	80 (11.8 %)	80 (10.7 %)
Internal medicine	125 (18.4 %)	135 (18.1 %)
Oncology	39 (5.7 %)	47 (6.3 %)
Orthopedic surgery	54 (8.0 %)	71 (9.5 %)
Urology	15 (2.2 %)	21 (2.8 %)
Ear, Nose, Throat	27 (4.0 %)	33 (4.4 %)
Other	3 (0.4 %)	4 (0.5 %)

**Table 2:** Female vs. male gender of the patient

	Female patient	Male patient	*p*-value
Case 1	39.9 %	39.0 %	n.s.
Case 2	37.9 %	38.3 %	n.s.
Case 3	40.4 %	43.9 %	n.s.
Case 4	70.7 %	68.1 %	n.s.
Case 5	63.0 %	62.0 %	n.s.
Case 6	66.9 %	69.8 %	n.s.
Case 7	48.9 %	53.3 %	n.s.
Case 8	65.6 %	63.6 %	n.s.

Proportion of patients deemed in need of ICU care dependent of patient gender

Female physicians tended to be more willing to admit patients than their male colleagues, regardless of the gender of the patient (Table 3). As seen in the table, this was statistically significant in cases 1, 5 and 6. In contrast, for case 7, male physicians were more willing than female physicians to admit a patient to the ICU. There was also a significant difference among different specialties, where anesthesiologists/intensive care physicians were more prone to admit patients than any other specialty in five out of eight cases (Table 4).

**Table 3:** Respondent: female vs. male, regardless of the gender of the patient

	Female respondent	Male respondent	*p*-value
Case 1	42.4 %	37.0 %	0.04
Case 2	37.1 %	38.9 %	n.s
Case 3	41.4 %	42.8 %	n.s.
Case 4	72.0 %	67.3 %	n.s.
Case 5	67.5 %	58.7 %	0.001
Case 6	73.5 %	64.4 %	<0.001
Case 7	47.4 %	54.1 %	0.01
Case 8	62.8 %	66.1 %	n.s.

**Table 4:** Respondent: Anesthesiology/intensive care vs. others, regardless of the gender of the patient

	Anesthesiology/intensive care	Others	*p*-value
Case 1	53.0 %	35.5 %	<0.001
Case 2	50.6 %	34.3 %	<0.001
Case 3	44.5 %	41.5 %	n.s.
Case 4	70.3 %	69.1 %	n.s.
Case 5	73.6 %	59.1 %	<0.001
Case 6	67.3 %	68.6 %	n.s.
Case 7	65.5 %	46.9 %	<0.001
Case 8	80.0 %	60.0 %	<0.001

## Discussion

Our key finding was that there were no significant differences in admittance to ICU with regards to patient gender. An underlying gender bias in Swedish hospital physicians could thus not be demonstrated. Anesthesiologists/intensivists were significantly more willing to admit patients to the ICU as compared to all other specialties. Female physicians also tended to admit more patients as compared to their male colleagues.

In a study where modified essay questions were distributed during a national examination for 239 Swedish interns significant gender differences were detected [6]. The examinees were allocated to suggest management of neck pain in a female or male. Laboratory tests were requested more often for males, whereas psychosocial questions, need for physiotherapist and drug prescriptions were more common for females. Using a similar methodology, our lack of significant differences based on gender in willingness to admit patients to the ICU, is encouraging. More than any other field of medicine, cardiac care has been scrutinized and found to be plagued by gender bias [7–9]. In fact, this issue was addressed back in 2006 in a statement from the policy conference of the European Society of Cardiology [10]. However, change does not happen overnight as illustrated by a press release in May 2014, from the World Heart Federation, where experts again called for an end to gender bias in cardiovascular disease [11]. Regarding critical care, one large cohort study of almost 19 000 patients in 98 ICUs from the US, Canada and Brazil revealed that females with severe sepsis/septic shock had a higher risk of dying in the hospital than males. This difference remained after multivariable adjustment. Significant gender disparities were also found in some aspects of care delivery, but these did not explain the higher mortality in female patients [12]. In a study from 2006 female sex was associated with issuance of do not resuscitate orders in patients receiving emergency surgery [13]. Significant gender-differences in ICU related processes of care have also been described elsewhere [1, 14, 15].

In above mentioned studies regarding the critically ill focus has been on ICU outcomes and ICU interventions. The purpose of this examination was to examine if gender bias concerning ICU admission exists. Do Swedish hospital physicians have different admission thresholds for males and females? In the present study physicians working in anesthesiology/intensive care as well as non-ICU-colleagues were included as the aim was to test a possible gender bias for ICU-admittance. In different ways, both intensivists- and non-intensivists set the bar for the ICU-threshold, i.e., unless the hospitalist first deems that the patient may be a candidate for intensive care and alerts the intensivist, no admission will take place. As mentioned in the introduction, in a trauma setting Gomez and co-workers examined almost 27 000 severely injured patients [3]. They found a significantly smaller proportion of females to receive trauma center care compared to males, an association that persisted after adjustment for confounders. The authors speculate that reasons for this differential access could be perceived difference in injury severity, likelihood of benefiting from trauma center care, or subconscious gender bias. In 2002 Raine et al. demonstrated a possible influence of patient gender on admission to intensive care [16]. Scrutinizing 46 587 admissions in 91 units across England, Wales, and Northern Ireland the authors found that certain conditions seemed to favor men, and other women. Inequity was found for patients with myocardial infarction and neurological bleeding, implying more restrictive admission criteria for women. In contrast, the authors found signs of gender bias against male ventricular failure patients, possibly explaining the higher male mortality in that group. In a register study from Finland, males had poorer ICU outcome as compared to women, whilst consuming approximately two-thirds of ICU resources [17]. Similar findings were found in Sweden regarding ICU consumption [2]. Considering the response rate in our study, and the fact that the largest specialist group was anesthesiologists/intensivists, there are a few things to consider in view of non-significant findings regarding ICU admission gender bias. Firstly, the anesthesiologists/intensivists admitted more patients overall, possibly due to expert knowledge on critically ill patients present, it could be speculated that expert knowledge counteracts gender bias. Specific and solid knowledge of a certain field is likely to limit the scope for basing evaluation on non-factual circumstances, such as preconceived ideas about men and women, i.e., gender norms. Secondly, female physicians were more willing to admit patients overall, regardless of gender. This is somewhat in line with other investigations, where female doctors have been found to adhere more to treatment guidelines [18]; and also to spend more time in consultation with female and male patients alike [19]. Lastly, the age distribution of the responders indicates that younger physicians are more willing than older colleagues to fill out surveys, and this may have contributed to the lack of significant findings.

This study has considerable strengths. It is unique in that it provides hospital physicians with two surveys, where eight potential patient cases are distributed, and where the only thing differing is the gender of the patient described. This allows for a detection of gender bias, removing a possible influence of next of kin. Moreover, the methodology allowed us to examine if different specialists made different choices. The cases were selected to be highly contentious - cases that would typically be admitted by approx 50 % and refused by 50 % of clinicians. As mentioned above, the fact that anesthesiologists/intensivists were more likely to admit patients based on the case descriptions may be attributable to their knowledge of how critical illness is presented in a ward- or emergency room patient. This is actually the first time the so called afferent limb, the mechanism by which medical emergency team responses are triggered, has been tested [20]. Another feature is that female- as compared to male responding physicians could be analyzed. The strength of the present study, i.e., presenting “pure” cases, has some drawbacks. In presenting physicians with written cases on a screen we remove factors that could contribute to decisions playing a paramount role in a real hospital setting. Now, our aim was not to investigate physicians’ ability to make perfectly adequate clinical judgements based on a paper case, but rather to find any differences in decision making that could only be attributed to the patient gender. Our study design is obviously associated with a number of limitations. One might speculate on who chooses to answer a survey like this; our response rate was around 30 %. Are we introducing a selection bias, where the certain types of doctors respond and others do not? Are we getting responses from empathic, young, non-biased colleagues who *even take the time to answer online surveys*? As mentioned, the age distribution indicates that younger physicians are overrepresented among responders. Despite having over 1400 physicians taking the survey, we lack power to test if gender bias is overrepresented with regards to age, medical specialty and/or combinations of respondent characteristics. With a (much) larger cohort, one could investigate if young female surgeons, old male internists or many other combinations differed in their admission choices. It is hard to say if our results are generalizable to other countries with other gender norms. Lastly, differences in ICU beds per capita and admitting rights to ICU could also make comparisons difficult.

## Conclusions

In the present study we could demonstrate an absence of a gender bias among Swedish hospital physicians regarding willingness to admit patients to ICU.

### Key messages

- Gender bias related to ICU admission could not be demonstrated among physicians working in Sweden but larger international studies are needed
- Anesthesiologists/intensivist were more likely to recommend ICU admission as compared to other specialists
- Female physicians were more likely to recommend ICU admission as compared to male physicians

## Additional file

Additional file 1:
**Supplementary data.** Has the full survey, case changes (**Table S1**) and participatinghospitals (**Table S2**). (DOCX 27 kb)

## Abbreviations

ICU: Intensive care unit
SIR: Swedish Intensive care Register
