# Supplementary material for: The influence of gender on ICU admittance
Source: Scand J Trauma Resusc Emerg Med. 2015 Dec 24;23:108. doi: 10.1186/s13049-015-0191-2 (PMC4690252; doi:10.1186/s13049-015-0191-2)
Supplement: Additional file 1: — Supplementary data. Has the full survey, case changes (Table S1) and participatinghospitals (Table S2). (DOCX 27 kb) [file 13049_2015_191_MOESM1_ESM.docx]

**Additional file 1. Cases (Survey 1, FMFFFMFM)**

**Case 1**

A 55 year old woman with metastatic hepatocellular cancer, cirrhosis and palliative oncological treatment (median survival 46 weeks) is treated on the infectious diseases ward for pneumonia.

She has become increasingly tachypnoeic and a nurse calls you and informs you that her respiratory rate is 32 breaths per minute

Saturation 90% on10l O2. Blood pressure and pulse normal.

Do you think this patient needs to be admitted to the intensive care unit?

Yes/No

**Case 2**

In the emergency department there is a 68 year old man with an acute exacerbation of chronic cardiac failure.

He has a history of hypertension and diabetes mellitus. A year ago he suffered an acute myocardial infarction which was treated with a stent in the circumflex artery. He has now had a period of depression with increased alcohol intake and tells you he hasn’t been taking his medications properly.

On examination he has conversational dyspnoea, central cyanosis, peripheral oedema and pulmonary crepitations. He has a raised jugular venous pulse, tender hepatomegaly and mild ascites. He denies chest pain and his ECG shows no arrhythmia or signs of ischemia and his cardiac enzymes are normal. His electrolytes, liver function tests, CRP and blood glucose are normal. His BNP/ProBNP levels are mildly elevated.

Do you think this patient needs to be admitted to the intensive care unit?

Yes/No

**Case 3**

A 66 year old woman presents to the emergency department feeling generally unwell. She lives alone and has no relatives.

Past medical history: Smoker, alcoholism, 2 liver transplants, severe pancreatitis with secondary diabetes, hypertension, renal failure with a baseline creatinine of approximately 250. She also has ischemic heart disease with a myocardial infarction 2 months ago, a stent in the LAD and a distal stenosis in the RCA. Echocardiography at that time showed an LVEF of 30%.

She presents with one week’s history of increasing tiredness, pain in her whole body and rigors. She has no urinary or respiratory symptoms, normal bowel movements and has taken all of her medications as normal.

She is awake and orientated, and afebrile. Respiratory rate 18/min, saturation 97% on air. Pulse 70, blood pressure initially 90/60 but falls after half an hour to 60/40, pulse unchanged.

She is treated with antibiotics and fluids and her blood pressure returns to 90 systolic.

Do you think this patient needs to be admitted to the intensive care unit?

Yes/No

**Case 4**

Case 3 continued

The patient has been admitted to the medical high dependency unit with continuous monitoring and 24 hours has passed.

Her creatinine is now 600 micromol/L (6.78mg/dL). A CT of her abdomen is normal. Her CRP is 75.

She is responding poorly to fluid resuscitation and is hypotensive again despite 6 litres of fluid.

Do you think this patient needs to be admitted to the intensive care unit?

Yes/No

**Case 5**

A 69 year old woman comes in to the resuscitation room in the emergency department.

She was found with a depressed level of consciousness on the floor at home, it is not known how long she had been lying there.

She has a previous history of hypertension, smoking, is overweight and has type 2 diabetes.

On arrival to the emergency department she is GCS 9 (opens eyes to pain, flexes upper limbs to pain and speaks individual words only)

Pulse 100/min, BP 205/90, breathing spontaneously with 95% saturations on 10l O2, blood glucose 14mmol/l.

CT shows a well demarcated infarct in the right middle cerebral artery territory

Do you think this patient needs to be admitted to the intensive care unit?

Yes/No

**Case 6**

63 year old man with COPD, home oxygen.

An inpatient on a medical ward for the last 3 days with reduced oxygen saturation, no signs of infection but treated anyway with antibiotics.

Despite full treatment the patient has not shown any signs of improvement.

The nurse informs you that his respiratory rate is now 36/min and his saturations are 82% on 2l of oxygen. His other vital signs are normal but the patient is tired and is becoming drowsy.

Do you think this patient needs to be admitted to the intensive care unit?

Yes/No

**Case 7:**

In the resuscitation room you see Berit, 72 years old, who has been brought in by ambulance after a fall downstairs. She is awake and can recount her medical history but is a little confused.

She has rapid breathing and 92% saturations on 2l of oxygen via nasal cannula. She complains of pain on deep breathing. Her blood pressure is 120/60 and her pulse is 100/minute.

Her abdomen is soft and non-tender and her pelvis feels stable.

There are no signs of limb fractures and her peripheral pulses are normal.

Pain x-rays of the chest and pelvis show multiple rib fractures, left 3rd-9th ribs and right 7th and 8th.

The CT scanner is unfortunately temporarily out of order and will likely be so for a further four hours.

Do you think this patient needs to be admitted to the intensive care unit?

Yes/No

**Case 8**

You are called from the surgical ward during the morning.

The nurse tells you about a patient who had a colectomy last week. He is now confused and disorientated. It started last night and the on-call doctor, who was in theatre, prescribed sleeping tablets and morphine.

The patient, a 64 year old man with diverticulitis, high blood pressure and diet controlled diabetes, only became more confused from the medications and has hardly slept at all.

When you see the patient he has pulled out her nasogastric tube and has vomited. He is agitated and breathing rapidly with a saturation of 89% on 5l of oxygen.

The patient has a fever of 39 degrees and a blood pressure of 110/70.

Do you think this patient needs to be admitted to the intensive care unit?

Yes/No

**Additional file 1: Table S1. Design of surveys**

|  | **Survey 1** | **Survey 2** |
| --- | --- | --- |
| **Case 1** | Female patient | Male patient |
| **Case 2** | Male patient | Female patient |
| **Case 3** | Female patient | Male patient |
| **Case 4** | Female patient | Male patient |
| **Case 5** | Female patient | Male patient |
| **Case 6** | Male patient | Female patient |
| **Case 7** | Female patient | Male patient |
| **Case 8** | Male patient | Female patient |

**Appendix Table S2. Participating hospitals**

Sahlgrenska University Hospital

Stockholm South General Hospital (Södersjukhuset)

Norra Älvsborg County Hospital in Trollhättan

Karolinska University Hospital

Karlstad Hospital

Uppsala University Hospital

Västmanland Hospital Västerås

Danderyd Hospital

Örebro University Hospital

Skåne University Hospital (Malmö and Lund)

University Hospital of Umeå

Linköping University Hospital

Södra Älvsborg Hospital

Skaraborg Hospital Skövde
